# Supplementary material for: Bone Deformities through the Prism of the International Classification of Functioning, Disability and Health in Ambulant Children with Cerebral Palsy: A Systematic Review
Source: Children (Basel). 2024 Feb 16;11(2):257. doi: 10.3390/children11020257 (PMC10888000; doi:10.3390/children11020257)
Supplement: Supplementary file 1 [file children-11-00257-s001.zip › Children_SM3.pdf]

Title	Author	Year	No bone variable	No function outcome	No correlation	Method evaluation	Included	Funding
Determinants of Hip and Femoral Deformities in Children With Spastic Cerebral Palsy.	Cho & al.	2018					X	None declared
Correlation between physical examination and three-dimensional gait analysis in the assessment of rotational abnormalities in children with cerebral palsy	Teixeira & al.	2018					X	None declared
Femoral anteversion assessment: Comparison of physical examination, gait analysis, and EOS biplanar radiography.	Westberry & al.	2018					X	No specific funding
Correlation of the torsion values measured by rotational profile, kinematics, and CT study in CP patients.	Kim & al.	2017					X	None declared
Correlation between transverse plan kinematics and foot progression angle in children with spastic diplegia	Presedo & al.	2017					X	None declared
Are clinical parameters sufficient to model gait patterns in patients with cerebral palsy using a multilinear approach?	Bonnefoy-Mazure & al.	2016	X					
The effect of postural control and balance on femoral anteversion in children with spastic cerebral palsy.	Karabicak & al.	2016					X	None declared
Gait pattern differences between children with mild scoliosis and children with unilateral cerebral palsy.	Domagalska-Szopa & al.	2014	X					
Discrimination of Abnormal Gait Parameters Due to Increased Femoral Anteversion from other Effects in Cerebral Palsy	Akalan & al.	2013			X			
Sit-to-stand movement in children with hemiplegic cerebral palsy: relationship with knee extensor torque and social participation.	Dos Santos & al.	2013	X					

Title	Author	Year	No bone variable	No function outcome	No correlation	Method evaluation	Included	Funding

Title	Author	Year	No bone variable	No function outcome	No correlation	Method evaluation	Included	Funding
Femoral anteversion and tibial torsion only explain 25% of variance in regression analysis of foot progression angle in children with diplegic cerebral palsy	Lee & al.	2013					X	Resarch funding (grant no. 02-2011-045) from Seoul National University Bundang Hospital, Republic of Korea
Are clinical measurements linked to the gait deviation index in cerebral palsy patients?	Sagawa & al.	2013	X					
Morphometric analysis of the femur in cerebral palsy: 3-dimensional CT study.	Gose & al.	2010		X				
Correlation Between Lower Limb Bone Morphology and Gait Characteristics in Children With Spastic Diplegic Cerebral Palsy:	Carriero & al.	2009			X			
Relationship between kinematic knee deviations and femoral anteversion in children with cerebral palsy	Piccinini & al.	2009			X			
Do dynamic and static clinical measurements correlate with gait analysis parameters in children with cerebral palsy?	Desloovere & al.	2006					X	None declared
The mid-point of passive hip rotation range is an indicator of hip rotation in gait in cerebral palsy.	Kerr & al.	2003					X	None declared
Effects of lower limb torsion on ankle kinematic data during gait analysis	Song & al.	2001				X		
Evaluation of rotational gait abnormality in the patients cerebral palsy.	Aktas & al.	2000					X	None declared
Femoral anteversion and neck-shaft angles in hip instability in cerebral palsy.	Laplaza & al.	1994		X				
Femoral torsion and neck-shaft angles in cerebral palsy.	Laplaza & al.	1993			X			
