# Supplementary material for: Bone Deformities through the Prism of the International Classification of Functioning, Disability and Health in Ambulant Children with Cerebral Palsy: A Systematic Review
Source: Children (Basel). 2024 Feb 16;11(2):257. doi: 10.3390/children11020257 (PMC10888000; doi:10.3390/children11020257)
Supplement: Supplementary file 1 [file children-11-00257-s001.zip › Children_SM4.pdf]

Title	Author	Year	SEMLS or including a soft tissu procedure	Non-ambulatory CP	No available outcome	Adult population	Included	Funding
Is Standing Function Improved After Orthopaedic Surgery in Children With Cerebral Palsy at GMFCS Levels III/IV?	Lennon & al.	2022	X					
Mid-Term Results of Distal Femoral Extension and Shortening Osteotomy in Treating Flexed Knee Gait in Children with Cerebral Palsy	Geisbüsch & al.	2022	X					
Genu Valgum After Distal Femur Extension Osteotomy in Children With Cerebral Palsy.	Liou & al.	2022	X					
Prone vs Supine Positioning for Femoral Derotation Osteotomy: Kinematic and Physical Examination Outcomes Suggest Both Can Achieve Desired Results	Givon & al.	2022	X					
What are the long-term outcomes of lateral column lengthening for pes planovalgus in cerebral palsy?	Kruger & al.	2022	X					
Femoral derotation osteotomy—Does intraoperative electromagnetic tracking reflect the dynamic outcome?	Geisbüsch & al.	2022	X					
Proximal femoral derotation osteotomy in children with CP : long term outcome and the role of age at time of surgery	Vermuyten & al.	2021	X					
Long-term functional outcomes after an external femoral derotation osteotomy in individuals with cerebral palsy	Boyer & al.	2021				X		
Kinematics after unilateral femoral derotation osteotomy in children with diplegic cerebral palsy	Desailly & al.	2020	X					
Double calcaneal osteotomy in treatment of flexible planovalgus foot deformity in ambulatory cerebral palsy. A case series study	Aly & al.	2019	X					
Orthopaedic Surgery in Dystonic Cerebral Palsy	Blumetti & al.	2019	X					
The long-term outcome of pelvic asymmetry during gait in children with cerebral palsy following unilateral femoral derotation osteotomy	Perotti &al.	2019	X					
Supracondylar femoral rotation osteotomy affects frontal hip kinetics in children with bilateral cerebral palsy	Thielen & al.	2019	X					
Long-Term Outcomes of Distal Femoral Extension Osteotomy and Patellar Tendon Advancement in Individuals with Cerebral Palsy	Boyer & al.	2018	X					
Preoperative hip rotation moments do not predict long-term development after femoral derotation osteotomy in children with cerebral palsy	Braatz & al.	2018	X					
The effect of hip reconstruction on gross motor function levels in children with cerebral palsy	Cobanoglu & al.	2018	X					
Changes in gait parameters after femoral derotational osteotomy in cerebral palsy patients with medial femoral torsion	Kim & al.	2018	X					
The increase of anterior pelvic tilt after crouch gait treatment in patients with cerebral palsy	Morais & al.	2018	X					
Proximal versus distal femoral derotation osteotomy in bilateral cerebral palsy	Niklasch & al.	2018	X					
Long-term development of overcorrection after femoral derotation osteotomy in children with cerebral palsy	Niklasch & al.	2018	X					
Surgical reconstruction of hip subluxation and dislocation in children with cerebral palsy	Oto & al.	2018	X					
Long-term outcomes over 10 years after femoral derotation osteotomy in ambulatory children with cerebral palsy	Sung & al.	2018	X					
Long-term outcomes of external femoral derotation osteotomies in children with cerebral palsy	Õunpuu & al.	2017	X					
Hallux valgus deformity correction without fusion in children with cerebral palsy	Bayhan & al.	2017	X					
Correlation of technical surgical goals to the GDI and investigation of post-operative GDI change in children with cerebral palsy	Bickley & al.	2017	X					

Changes in hip abductor moment 3 or more years after femoral derotation osteotomy among individuals with cerebral palsy	Boyer & al.	2017					X	Gait and Motion Outcomes Fund of the Gillette Children's Foundation
Abnormality of standing posture improves in patients with bilateral spastic cerebral palsy following lower limb surgery	Buddhdev & al.	2017	X					
Persistence and Recurrence Following Femoral Derotational Osteotomy in Ambulatory Children With Cerebral Palsy	Church & al.	2017	X					
Long-term Outcome of External Tibial Derotation Osteotomies in Children With Cerebral Palsy	Er & al.	2017	X					
Long-term Outcome of Internal Tibial Derotation Osteotomies in Children With Cerebral Palsy	Er & al.	2017	X					
Hip Reconstruction in Children With Unilateral Cerebral Palsy and Hip Dysplasia	Abousamra & al.	2016	X					
Satisfaction and pain levels after proximal femoral valgus osteotomy according to Schanz in patients with cerebral palsy and hip dislocation	Harmsen & al.	2016		X				
Salvage procedures for the painful chronically dislocated hip in cerebral palsy	Hwang & al.	2016		X				
The influence of timing of knee recurvatum on surgical outcome in cerebral palsy	Klotz & al.	2016	X					
Outcomes of Orthopaedic Surgery With and Without an External Femoral Derotational Osteotomy in Children With Cerebral Palsy	McMulkin & al.	2016	X					
Correction of gait after derotation osteotomies in cerebral palsy: Are the effects predictable?	Böhm & al.	2015	X					
Asymmetric pelvic and hip rotation in children with bilateral cerebral palsy: uni- or bilateral femoral derotation osteotomy?	Niklasch & al.	2015	X					
Superior functional outcome after femoral derotation osteotomy according to gait analysis in cerebral palsy	Niklasch & al.	2015	X					
Long-term results and outcome predictors in one-stage hip reconstruction in children with cerebral palsy	Rutz & al.	2015	X					
Gait improvement surgery in ambulatory children with diplegic cerebral palsy	Terjesen & al.	2015	X					
Long-term ambulatory change after lower extremity orthopaedic surgery in children with cerebral palsy: a retrospective review	Yu & al.	2015	X					
Crouch gait changes after planovalgus foot deformity correction in ambulatory children with cerebral palsy	Kadhim & al.	2014	X					
Do changes in torsional magnetic resonance imaging reflect improvement in gait after femoral derotation osteotomy in patients with cerebral palsy?	Braatz & al.	2013	X					
Does the level of proximal femur rotation osteotomy influence the correction results in patients with cerebral palsy?	De Morais & al.	2013	X					
Medial column stabilization improves the early result of calcaneal lengthening in children with cerebral palsy	Huang & al.	2013			X			
Long-term outcome of planovalgus foot surgical correction in children with cerebral palsy	Kadhim & al.	2013	X					
Outcomes of correction of internal hip rotation in patients with spastic cerebral palsy using proximal femoral osteotomy	De Morais & al.	2012	X					
Hip resurfacing with femoral osteotomy for painful subluxed or dislocated hips in patients with cerebral palsy	Prosser & al.	2012				X		
The effects of femoral derotation osteotomy in cerebral palsy: a kinematic and kinetic study	Cimolin & al.	2011					X	No specific funding

Modified Goal Attainment Scale outcomes for ambulatory children: with and without orthopedic surgery	Gordon & al.	2011	X					
Surgical management of hip subluxation and dislocation in children with cerebral palsy: isolated VDRO or combined surgery?	Huh & al.	2011			X			
Combined femoral and pelvic osteotomies versus femoral osteotomy alone in the treatment of hip dysplasia in children with cerebral palsy	Al-Ghadir & al.	2009		X				
The effects of femoral derotation osteotomy in children with cerebral palsy: an evaluation using energy cost and functional mobility	Amichai & al.	2009	X					
Calcaneal lengthening for planovalgus deformity in children with cerebral palsy	Etti & al.	2009	X					
Effects of orthopedic intervention in adolescents and young adults with cerebral palsy	Westwell & al.	2009	X					
The outcome of surgical intervention for early deformity in young ambulant children with bilateral spastic cerebral palsy	Gough & al.	2008	X					
Results of treatment when orthopaedic surgeons follow gait-analysis recommendations in children with CP	Lofterød & al.	2008	X					
Changes in dynamic foot pressure after surgical treatment of valgus deformity of the hindfoot in cerebral palsy	Park & al.	2008	X					
Femoral derotation osteotomy in cerebral palsy: precise determination by tables	Cobeljić & al.	2006	X					
Subtrochanteric valgus osteotomy for chronically dislocated, painful spastic hips	Hogan & al.	2006	X					
Leg lengthening as part of gait improvement surgery in cerebral palsy: an evaluation using gait analysis	Saraph & al.	2006	X					
Results of tibial rotational osteotomy without concomitant fibular osteotomy in children with cerebral palsy	Ryan & al.	2005	X					
Results of tibial rotational osteotomy without concomitant fibular osteotomy in children with cerebral palsy	Ryan & al.	2005	X					
Calcaneal lengthening for the planovalgus foot deformity in children with cerebral palsy	Yoo & al.	2005	X					
Spastic hemiplegic cerebral palsy and the femoral derotation osteotomy: effect at the pelvis and hip in the transverse plane during gait	Aminian & al.	2003	X					
Comparison of proximal and distal rotational femoral osteotomy in children with cerebral palsy	Kay & al.	2003	X					
Proximal femoral varus rotation osteotomy in cerebral palsy: a prospective gait study	Murray-Weir & al.	2003	X					
Femoral derotation osteotomy in spastic diplegia. Proximal or distal?	Pirpiris & al.	2003	X					
Rehabilitation after femoral osteotomy in cerebral palsy	Stasikelis & al.	2003	X					
Long-term effects of femoral derotation osteotomies: an evaluation using three-dimensional gait analysis	Õunpuu & al.	2002	X					
Propulsive function during gait in diplegic children: evaluation after surgery for gait improvement	Zwick & al.	2001	X					
The severely unstable hip in cerebral palsy. Treatment with open reduction, pelvic osteotomy, and femoral osteotomy with shortening	Root & al.	1995	X					
Treatment of the chronically dislocated hip in adolescents with cerebral palsy with femoral head resection and subtrochanteric valgus osteotomy	McHale & al.	1990	X					
Surgery for hip dislocation in cerebral palsy	Bos & al.	1987	X					
