# Supplementary material for: Bone Deformities through the Prism of the International Classification of Functioning, Disability and Health in Ambulant Children with Cerebral Palsy: A Systematic Review
Source: Children (Basel). 2024 Feb 16;11(2):257. doi: 10.3390/children11020257 (PMC10888000; doi:10.3390/children11020257)
Supplement: Supplementary file 1 [file children-11-00257-s001.zip › Children_SM_1.pdf]

## Supplementary Material 1: Search strings used in the systematic review for each question

| | Population | Bone morphology | Surgery | Body functions | Activity | Participation | Study type |
|---|---|---|---|---|---|---|---|
| Question 1 | Cerebral Pals* | Bone* OR deform* OR morpholog* OR shape* OR femur OR femor* OR tibia* OR foot OR torsion* OR rotation* OR anteversion OR tilt OR obliquity OR "bicondylar angle" OR "neck-shaft angle" "lower limb" OR "Bone and Bones"[Mesh] OR "Bone Anteversion"[Mesh] OR "Bone Malalignment"[Mesh] OR "Bone Development"[Mesh] OR "Pelvic Bones"[Mesh] OR "Leg Bones"[Mesh] OR "Hip"[Mesh] OR "Hip Joint"[Mesh] OR "Femur"[Mesh] OR "Femur Neck"[Mesh] OR "Femur Head"[Mesh] OR "Tibia"[Mesh] OR "Torsion Abnormality"[Mesh] OR "Foot Deformities"[Mesh] | | gait OR "motor capacity" OR "motor capability" OR "range of motion" OR "ROM" OR strength OR spasticity OR "motor control" OR "selectivity motor control" OR speed OR balance OR "postural control" OR "TUG" OR "Time* Up and Go" OR "walk* test*" OR "Gait Disorders, Neurologic"[Mesh] OR "Mobility Limitation"[Mesh] OR "Walk Test"[Mesh] OR "Postural Balance"[Mesh] OR "Walking Speed"[Mesh] OR "Range of Motion, Articular"[Mesh] OR "Biomechanical Phenomena"[Mesh] OR "Muscle | activit* OR walk* OR ambulat* OR stand* OR run* OR jump*OR "daily life" OR "functional disabilit*" OR mobility OR performance OR OR Walk* OR Gait OR Ambulat* OR Gait speed OR Walk* perimeter OR Stand OR Run OR Jump OR "6-Minute walk test" OR "Endurance shuttle walk test" OR "ESWT" OR "Incremental shuttle walk test" OR "ISWT" OR Walking[Mesh] OR"Activities of Daily Living"[Mesh] | Participation OR school OR leisure* OR game* OR Hobb* * OR Play* OR Sport* OR "Social Participation"[Mesh] OR "Leisure Activities"[Mesh] OR "Quality of life" [MeSH] | correlation* OR association* OR predict* OR regression* OR relationship* OR "multivariable analysis" |

| | | | | | | | |
|---|---|---|---|---|---|---|---|
| **Question 2** | Cerebral Pals* | Bone* OR deform* OR morpholog* OR shape*<br>OR femur OR femor*<br>OR tibia* OR foot<br>OR torsion* OR rotation* OR anteversion OR tilt OR obliquity<br>OR "bicondylar angle"<br>OR "neck-shaft angle" "lower limb" OR "Bone and Bones"[Mesh] OR "Bone Anteversion"[Mesh] OR "Bone Malalignment"[Mesh]<br>OR "Bone Development"[Mesh]<br>OR "Pelvic Bones"[Mesh]<br>OR "Leg Bones"[Mesh]<br>OR "Hip"[Mesh] OR "Hip Joint"[Mesh]<br>OR "Femur"[Mesh] OR "Femur Neck"[Mesh]<br>OR "Femur Head"[Mesh]<br>OR "Tibia"[Mesh]<br>OR "Torsion Abnormality"[Mesh]<br>OR "Foot Deformities"[Mesh] | Osseous reconstruction intervention OR Bon* surgery OR Osteotom* OR Operative treatment OR Orthopaedic surgery<br>OR Extension* osteotomy<br>OR Derotation* osteotomy<br>OR Rotation* osteotomy<br>OR Lengthen* osteotomy<br>OR Varisation osteotomy<br>OR Naviculectomy<br>OR "Surgery" [MeSH]<br>OR "Orthopedics" [MeSH]<br>OR "Osteotomy" [MeSH]<br>OR "Arthrodesis" [MeSH]<br>OR "Bone lengthening" [MeSH]<br>OR "Arhroplasty" [MeSH] ? | gait<br>OR "motor capacity"<br>OR "motor capability"<br>OR "range of motion" OR "ROM"<br>OR strength OR spasticity OR "motor control" OR "selectivity motor control"<br>OR speed<br>OR balance<br>OR "postural control"<br>OR "TUG" OR "Time* Up and Go"<br>OR "walk* test*"<br>OR "Gait Disorders, Neurologic"[Mesh]<br>OR "Mobility Limitation"[Mesh]<br>OR "Walk Test"[Mesh]<br>OR "Postural Balance"[Mesh]<br>OR "Walking Speed"[Mesh]<br>OR "Range of Motion, Articular"[Mesh]<br>OR "Biomechanical Phenomena"[Mesh]<br>OR "Muscle | activit* OR walk* OR ambulat*<br>OR stand* OR run* OR jump*OR "daily life" OR "functional disabilit*"<br>OR mobility OR performance OR<br>OR Walk* OR Gait OR Ambulat*<br>OR Gait speed<br>OR Walk* perimeter<br>OR Stand OR Run OR Jump<br>OR "6-Minute walk test" OR "Endurance shuttle walk test" OR "ESWT"<br>OR "Incremental shuttle walk test" OR "ISWT"<br>OR Walking[Mesh]<br>OR"Activities of Daily Living"[Mesh] | Participation OR school<br>OR leisure*<br>OR game*<br>OR Hobb*<br>* OR Play*<br>OR Sport* OR "Social Participation"[Mesh]<br>OR "Leisure Activities"[Mesh] OR "Quality of life" [MeSH] | correlation*<br>OR association*<br>OR predict*<br>OR regression*<br>OR relationship*<br>OR "multivariable analysis" |
