# Supplementary material for: Bone Deformities through the Prism of the International Classification of Functioning, Disability and Health in Ambulant Children with Cerebral Palsy: A Systematic Review
Source: Children (Basel). 2024 Feb 16;11(2):257. doi: 10.3390/children11020257 (PMC10888000; doi:10.3390/children11020257)
Supplement: Supplementary file 1 [file children-11-00257-s001.zip › Children_SM_2.pdf]

Supplementary Material 2: Quality evaluation of the studies included using the checklist for case series (CSS) for question 1 (Table A1) and by the Quebec quality assessment grid for question 2 (Table B1). Additional questions added for the review are shown in tables A2 and B2.

**A1** **Checklist for Case Series**	**Teixeira et al. 2018**	**Westberry et al. 2018**	**Cho et al. 2018**	**Presedo et al. 2017**	**Kim et al. 2017**	**Karabicak et al. 2016**	**Lee et al. 2013**	**Desloovere et al. 2006**	**Kerr et al. 2003**	**Aktas et al. 2000**
1. Were there clear criteria for inclusion in the case series?	Yes	Unclear	Unclear	Yes	Unclear	Yes	Yes	Yes	Unclear	No
2. Was the condition* measured in a standard, reliable way for all participants included in the case series?	No	No	No	No	No	No	No	No	No	No
3. Were valid methods used for identification of the condition* for all participants included in the case series?	No	No	No	No	No	No	No	No	No	No
4. Did the case series have consecutive inclusion of participants?	Yes	Yes	Yes	Unclear	Unclear	Unclear	Yes	No	Unclear	Unclear
5. Did the case series have complete inclusion of participants?	No	Yes	Yes	Unclear	Unclear	Yes	Yes	No	Unclear	Unclear
6. Was there clear reporting of the demographics of the participants in the study?	Unclear	Yes	Unclear	Unclear	Unclear	Yes	Unclear	Unclear	Unclear	Unclear
7. Was there clear reporting of clinical information of the participants?	Yes	Unclear	Yes	Yes	Unclear	Yes	Yes	Yes	No	No
8. ** Was the measure of bone morphology clearly reported?	Yes	Yes	Yes	Yes	Yes	Yes	Yes	Yes	Yes	Yes
9. Was there clear reporting of the presenting site(s)/clinic(s) demographic information?	Yes	Yes	Unclear	Unclear	No	Yes	Yes	Yes	Yes	Unclear
10. Was statistical analysis appropriate?	Yes	Unclear	Unclear	Yes	No	Unclear	Unclear	Unclear	Unclear	No

*Condition = Cerebral palsy
**Original question: Were the outcomes or follow up results of cases clearly reported?

**A2**<br><br>**Additional items for Research question 1**	Teixeira et al. 2018	Westberry et al. 2018	Cho et al. 2018	Presedo et al. 2017	Kim et al. 2017	Karabicak et al. 2016	Lee et al. 2013	Desloovere et al. 2006	Kerr et al. 2003	Aktas et al. 2000
i. Was the validity of measure of bone morphology clearly reported?	No	Yes	No	Unclear	Unclear	Unclear	Unclear	Unclear	Unclear	Yes
ii. Was the measure of body functions/activity/participation clearly reported?	Yes	Yes	Yes	Yes	Yes	Yes	Yes	Yes	Yes	Yes
iii. Was the validity of measure of body functions/activity/participation clearly reported?	Unclear	Yes	Unclear	Yes	No	Unclear	Yes	Unclear	Unclear	No
iv.Was the description of correlation analysis criteria reported?	Yes	Yes	No	No	No	Yes	No	Yes	No	No

B

Quality assessment grid for observational studies

	**Author**	Boyer et al.				Cimolin et al.			
	**Year**	2017				2011			
		Yes	No	Unclear	NA	Yes	No	Unclear	NA
**AIM**									
O1	Is the aim of the article stated by specifying, at a minimum, the intervention, the population and the main outcome?	X				X			
**METHOD**									
M1	Is the context of the study (location, recruitment period) clearly described?		X				X		
M2	Are the inclusion / exclusion criteria for study participants specified?	X				X			
M3	Is the method of recruiting participants adequate?	X						X	
M4	Is the targeted intervention sufficiently described (dosage, mode of administration, provider, other parameters)?		X			X			
M5	Is the comparator sufficiently described (dosage, mode of administration, provider, other parameters)?		X				X		
M6	Are the outcomes well defined?	X				X			
M7	Are all the measurement tools used standardized, valid and reliable?			X		X			
M8	Was the exposure or procedure without the knowledge of those evaluating the results?				X				X
M9	Are the planned statistical analyses appropriate?	X				X			
M10	Are precision measures such as confidence intervals, standard deviations or interquartile ranges planned?			X				X	
M11	Is an estimate of the number of participants needed to ensure adequate power made?		X				X		
**RESULTS**									
R1	Is the study population representative of the target population?	X					X		
R2	Is the number of participants analyzed sufficient to ensure the statistical power of the study for the assessment of the primary indicator?			X			X		
R3	Is the level of participation sufficient?				X				X
R4	Is the number of participants reported for each stage of the study (number at enrolment, eligibility, included in the study, having completed follow-up and included in the final analysis) ?	X				X			

R5	Is the percentage of lost subjects less than 20%?				X				X
R6	Are the reasons for lost to follow up identified?		X						X
R7	Are the lost to follow up subjects and participants compared?				X				X
R8	Are the characteristics of the study population sufficiently described?			X				X	
R9	Do the results presented take into account potentially confounding factors?		X					X	
R10	Is adherence to treatment* (compliance) assessed?		X				X		
R11	Are the analyses performed as planned?			X				X	
R12	Is the duration of follow-up adequate to observe the result?	X						X	
**DISCUSSION**									
D1	Do the findings of the study address the main objectives?	X				X			
D2	Do the authors identify the limits of the study?	X				X			
D3	Is the consistency of the results with those of other studies discussed?	X				X			
D4	Are the study findings consistent with the key findings?	X				X			
**OTHER CONSIDERATIONS**									
A1	Is the funding for the study reported?			X		X			
A2	Are conflicts of interest addressed?	X				X			
A3	Is there a potential for conflicts of interest?				X				X
The general quality of the study is:		Satisfactory				Satisfactory			
*post-operative treatment was considered									

B2

		Author	Boyer et al.				Cimolin et al.			
		Year	2017				2011			
**Additional items for Research question 2**			Yes	No	Unclear	NA	Yes	No	Unclear	NA
i.	Is the postoperative management protocol described?			X			X			
ii.	Are the characteristics of the control population sufficiently described?			X					X	
